# *Caridina sinanensis*, a new species of stygobiotic atyid shrimp (Decapoda, Caridea, Atyidae) from a karst cave in the Guizhou Province, southwestern China

**DOI:** 10.3897/zookeys.1008.54190

**Published:** 2020-12-30

**Authors:** Da-Jian Xu, Deng-Xu Li, Xiao-Zhuang Zheng, Zhao-Liang Guo

**Affiliations:** 1 College of Animal Science and Technology, Hunan Agricultural University, Changsha 410128, Hunan Province, China Hunan Agricultural University Changsha China; 2 Changsha Agricultural Comprehensive Administrative Law Enforcement Bureau, Changsha 410013, Hunan Province,China Changsha Agricultural Comprehensive Administrative Law Enforcement Bureau Changsha China; 3 Department of Animal Science, School of Life Science and Engineering, Foshan University, Foshan 528231, Guangdong Province, China Foshan University Foshan China

**Keywords:** *
Caridina
*, COI and 16S rRNA, freshwater biodiversity, karst landform, southwestern China, spelaeology, taxonomy

## Abstract

From a biodiversity survey of a subterranean habitat near Sinan County, Guizhou Province, southwestern China, a new atyid shrimp of the genus *Caridina* H. Milne Edwards, 1837, *C. sinanensis***sp. nov.** was discovered. The new species can be separated from other congeners based on a combination of characters including depigmentation in body and reduction of eyes, small pigment spot at the centre of the cornea, the shape of rostrum and the endopod of the 1^st^ male pleopod, and the relatively longer appendix interna on the appendix masculina of the 2^nd^ pleopod. Mitochondrial COI and 16S rRNA gene sequences also support the establishment of the new species. Information on the habitat, and the levels of threat are discussed to guide the conservation of *C. sinanensis***sp. nov.**

## Introduction

China’s karst landforms, accounting for about a third of the territory, are one of the largest and most spectacular karst sceneries in world (Shui et al. 2015). In 2007, the China Southern Karst Region was established as a Natural World Heritage site by the UNESCO World Heritage Committee during the 31^st^ World Heritage Conference held in Christchurch, New Zealand (Hou and Sun 2011). The extensive cave systems underlying the karst region harbor a vast variety of freshwater organisms, including atyid shrimp. Subterranean species are astonishing and bizarre outcomes of evolution over eons, either through regression or vicariance under natural selection; they have evolved to fully adapt to aquatic subterranean habitats, and they tend to exhibit conspicuous morphological adaptations and localized endemism (Culver et al. 2008). They are typically colorless, with reduced eyes, long antennae and ambulatory appendages (Mejía-Ortíz et al. 2006). More than 500,000 caves have been documented in China (Ran and Yang 2015), but only a small fraction of theses have been investigated. Knowledge on the distribution, abundance, life history, and ecology of atyid shrimp is inadequate, while the study of the cave-dwelling fauna remains in its initial stages. So far, 23 cave-dwelling atyid species are known in China from four genera of the family Atyidae: *Caridina* H. Milne Edwards, 1837, *Mancicaris* Liang, Guo & Tang, 1999, *Neocaridina* Kubo, 1938, and *Typhlocaridina* Liang & Yan, 1981. The majority of these species belong to the genus *Caridina*. Most cave atyids are highly specialized, phylogenetically unique, with restricted distributions and specialized habitat requirements. The epigean species, *Neocaridina palmata*, *Macrobrachium nipponense* and *M. superbum* are capable of surviving and reproducing in the caves.

During an inventory to evaluate the status of a variety of cave fauna along karst cave systems of Guizhou province, southwestern China in 2019–2020, specimens of atyid shrimp were collected from a cave river near Tangtou Town, Sinan County. After detailed examination of these specimens based on a combination of morphological and molecular features (COI and 16S rRNA), we are confident that our specimens have sufficient differences from known species to be recognized as a new species, *Caridina sinanensis* sp. nov. The present work provides detailed description, illustrations, molecular evidence, standardized diagnoses, colour photographs, and habitat information, as well as a distribution map. In addition, the conservation significance of this species is also briefly described. This raises the total number of subterranean atyid shrimp species known to date from China to 24 species.

## Materials and methods

### Cave description

**Figure 1.**
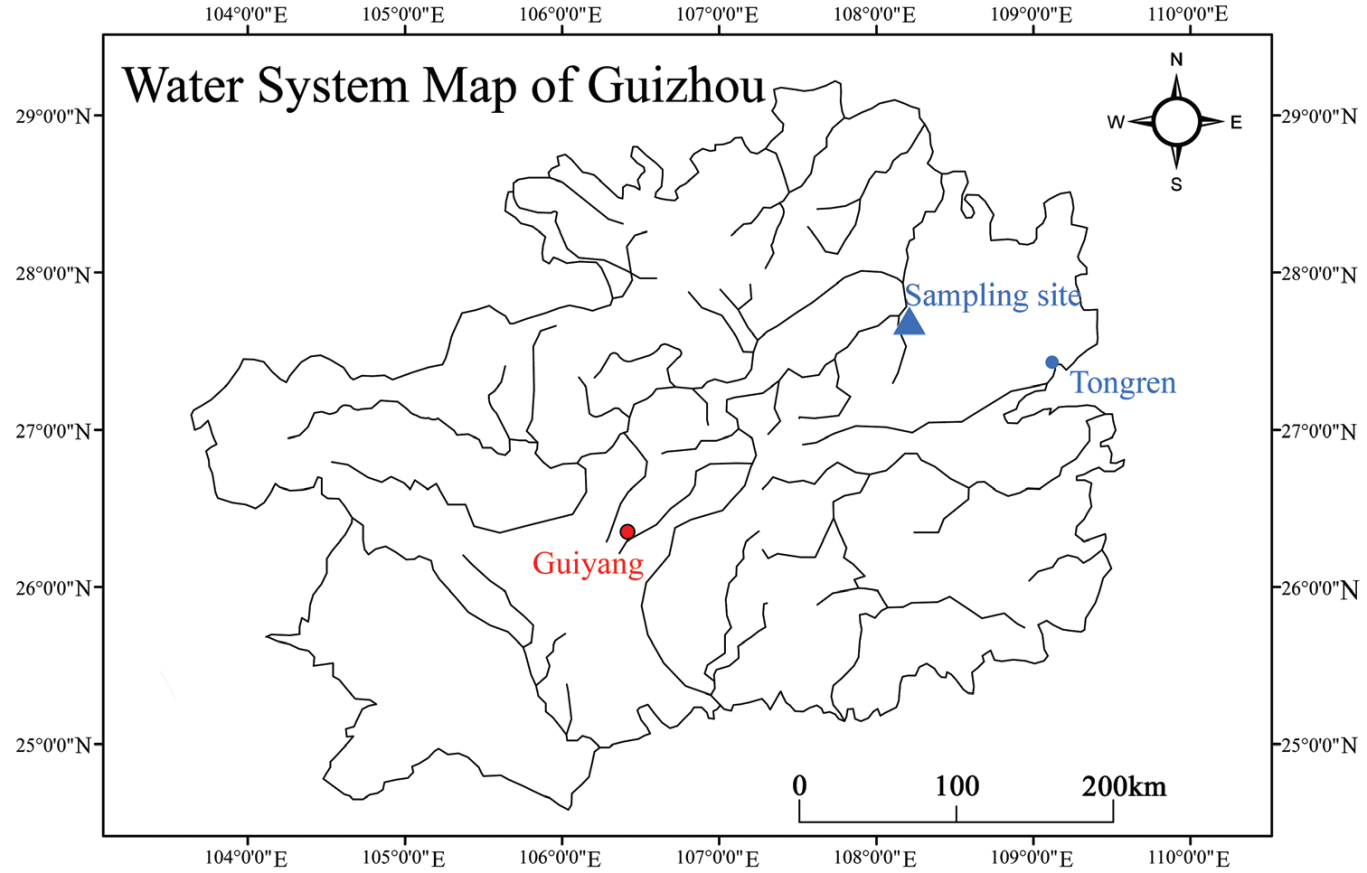
Map indicating rivers in Guizhou Province, China, with a triangle showing the sample site for *Caridina sinanensis* sp. nov.

**Figure 2.**
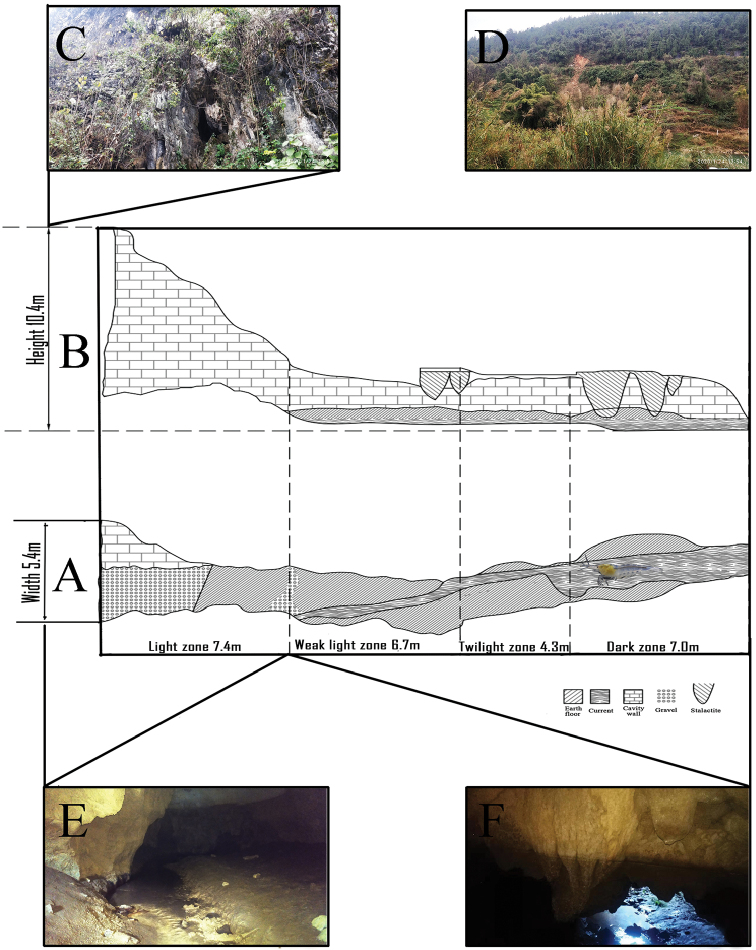
Schematic diagram of the cave **A** plan view of the cave **B** left profile view of the cave **C** close-up view of cave entrance **D** the vegetation around the cave **E** light zone of cave **F** dim light zone of cave.

The cave where the specimens were sampled is located in Pengjiaao, Tangtou Town, Sinan County, Guizhou Province, southwestern, China, at 27°44'10"N, 108°11'58"E (Fig. 1). The straight-line distance between the cave entrance and Provincial Road S203 is 6.4 m. The mouth of the cave is in a cliff along the north bank of the Shiqian River and at an elevation of 294.7 m. There is a dry artificial channel, 0.5 m in width and 0.8 m in height, in front of the cave entrance. Bare shale above the cave entrance is surrounded by scattered ferns, bryophytes, and vines. The arched opening of the cave is at the bottom of a crack in the substrate, 5.4 m in width and 10.4 m in height. The north oriented horizontal passage with a gravel and rocky floors extends to a hall about 80 m from the entrance. The passage lengths along the transect extending into the cave from the entrance were the light zone, weak light zone, twilight zone, and dark zone were 7.4, 6.7, 4.3, and 7.0 m, respectively. Two closely spaced conical stalactites are on the cave ceiling at 14 m from the cave’s entrance, and three more at 21 m from the entrance. A widening river appears in the middle of the horizontal passage 8 m into the cave. The drop between the surface of river and the entrance of cave is about 1 m. According to the local residents, heavy rains causes flooding with river water flowing out from cave. At ordinary times, the water was quite transparent, with sediment containing fine particulate matter, seeds, and fragments of leaves. Under the light of the headlights, dozens of shrimps were observed swimming or clinging to the bottom. The water depth is about 0.3–1.5 m, but the far wall was undercut and sloped precipitously to unknown depths. Only 20 m of the river is accessible, as the top of the cave intersects the water surface. The geomorphological features of the cave are shown in Fig. 2.

### Habitat description

Geological coordinates and basic hydrological and physicochemical parameters of the cave (width, depth, temperature, humidity, pH, CO, CO_2_, O_2_, H_2_S, and dissolved oxygen) were measured with the following instruments: Bosch GLM-30 Laser rangefinder, eTrex Venture GPS locator, JWSA2-2 temperature and hygrometer, LB-MS4X portable gas detector, BDO820 portable dissolved oxygen meter, and BPH-220 pH meter. The environmental physicochemical parameters of the cave are shown in Table 2, while the vegetation around the cave entrance and habitat features of cave interior are displayed in Fig. 2.

### Samples collection

We visited the cave three times, in January and March 2019 and February 2020. Each time some dozens of specimens were observed, and altogether 40 specimens were collected. Samples were collected with a sturdy, long-handled, fine-meshed dip net (mesh size 0.6 mm) and with the aid of headlamps. The sampling procedure was recorded with photographs and video-recordings. Specimens were placed in oxygenated polythene bags, anaesthetized with ice, and transported to the hotel, to be photographed, and fixed in 95% ethanol. Ethanol was changed after 24 h with fresh 75% ethanol.

### Morphological analysis

Specimens were examined using a dissecting microscope (Olympus SZX7). Illustrations and morphometric measurement of selected characters were recorded using a digital camera (DP22) mounted on a stereomicroscope (Olympus SZX7) and Olympus CellSens Entry v. 1.18 software.

The following abbreviations are used throughout the text: alt (altitude), cl (carapace length, measured from the postorbital margin to the posterior margin of the carapace), rl (rostral length, measured from the rostral tip to the postorbital margin), and tl (total length, measured from the rostral tip to the posterior margin of the telson). All measurements are in millimeters.

Specimens were deposited in the Department of Animal Science, School of Life Science and Engineering, Foshan University (FU), Guangdong, China.

### Molecular data collection and analysis

The abdominal muscle of the specimens were used for DNA extraction with an EasyPure Genomic DNA Kit (TransGen Biotech, Beijing, China) and then stored in a -20 °C freezer. To construct the molecular phylogeny of selected *Caridina* species, two mitochondrial gene fragments, 710 bp of the cytochrome oxidase subunit I (COI) and approximately 560 bp of the large ribosomal subunit (16S) were amplified and sequenced on an Applied Biosystems 3730 Analyzer (Applied Biosystems, Foster City, CA, USA) using COI primers LCO1490 and HCO2198 (Folmer et al. 1994), and 16S rRNA primers 16S-F-Car and 16S-R-Car1 (von Rintelen et al. 2007). All new sequences have been deposited in GenBank and the remaining sequences were downloaded from GenBank (Klotz and von Rintelen 2014) (Table 1).

**Table 1.** Species used in the molecular analysis, with details on sampling locations, GenBank accession numbers (COI, 16S rRNA).

Species	Sampling locality	GenBank accession numbers	
		COI	16S rRNA
*C. breviata*	China, from type locality	KP168788	KP168718
	China, from type locality	KP168789	KP168719
*C. cantonensis*	Qingyuan, China	KP168802	KP168720
	Qingyuan, China	KP168803	KP168721
*C. huananensis*	Yingde, Qingyuan	MN701607	MT446452
	Yingde, Qingyuan	MN701608	MT446453
*C. lanceifrons*	Dongfang, Hainan	MN701605	MT446450
	Dongfang, Hainan	MN701606	MT446451
*C. mariae*	Nankun Mountain, Huizhou	MN701601	MT446456
	Nankun Mountain, Huizhou	MN701602	MT446457
*C. nanaoensis*	China	KP168792	KP168755
*C. serrata*	Dong’ao Island, Zhuhai	MN701595	MT446454
	Dong’ao Island, Zhuhai	MN701596	MT446455
*C. sinanensis*	Sinan Guizhou	MT433962	MT434873
	Sinan Guizhou	MT433963	MT434874
	Sinan Guizhou	MT433964	MT434875
*C. trifasciata*	Zhuhai China	KP168795	KP168765
	Zhuhai China	KP168796	KP168766
*C. zhujiangensis*	Dong’ao Island, Zhuhai	MN701603	MT446448
	Dong’ao Island, Zhuhai	MN701604	MT446449
*N. palmata*	Yangshan, Qingyuan	MN701611	–
	Yangshan, Qingyuan	MN701611	–
	China	–	KP168779
	Hong Kong, China	–	KP168780

As there are no *Caridina longshan* and *C. alu* sequences on the GenBank, and no *C. longshan* and *C. alu* specimens were collected, only 22 sequences in this paper were analyzed. It is mainly based on nine species of *Caridina* shrimp from China, so as to conduct molecular analysis with *C. sinanensis* sp. nov. All sequences were aligned with MAFFT v. 7.037 software using the auto strategy and normal alignment mode (Katoh and Standley 2013). Appropriate models of sequence evolution were selected using ModelFinder (Kalyaanamoorthy et al. 2017), and consequently the GTR+F+I+G4 (COI) and the HKY+F+G4 (16S rRNA) were employed. Phylogenetic tree was constructed by Bayesian inference approach using MrBayes v. 3.2.6 (Ronquist et al. 2012), with two parallel runs, 2000000 generations, in which the initial 25% of sampled data were discarded as burn-in. Genetic distances were calculated using the Kimura 2-parameter model in MEGA v. 7.0 based on COI and 16S rRNA, respectively (Kumar et al. 2016).

## Results

### Taxonomy

#### Systematic accounts

**Family Atyidae De Haan, 1849**

#### Genus *Caridina* H. Milne Edwards, 1837

##### Caridina sinanensis sp. nov.

Taxon classificationAnimaliaDecapodaAtyidae

7FB1682D-ED04-5D47-8447-CF2682C14F28

http://zoobank.org/AC2E06AB-1DFF-49D6-8D76-26DB850E7597

Figs 3 , 4 , 5

###### Material examined.

***Holotype***: Adult male (FU, 2019-01-25-01), tl 16.7 mm, cl 4.8 mm, rl 1.5 mm; a cave river at Pengjiaao, Tangtou Town, Sinan County, Guizhou Province, southwestern, China (27°44'10"N, 108°11'58"E, alt. 294.7 m), 25 Jan. 2019. ***Paratypes***: 1 male (FU, 2019-01-25-02) cl 5.4 mm; 1 male (FU, 2019-01-25-03) cl 6.8 mm; 1 male (FU, 2019-01-25-04) cl 4.8 mm; 2 males (FU, 2019-01-25-05), cl 4.2–6.2 mm; 20 females (9 ovigerous) (FU, 2019-01-25-05), cl 4.9–6.6 mm, sampled together with the holotype.

###### Comparative material examined.

*Caridina semiblepsia* Guo, Choy & Gui, 1996. Adult male (FU, 1994-05-17-01), tl 17.5 mm, cl 4.5 mm, rl 0.7 mm; a cave river at Tongpatong, Baojing County, Hunan Province, China, 17 May 1994. Paratypes: 4 males (FU, 1994-05-17-02) cl 4.8–5.6 mm; 5 females (2 ovigerous) (FU, 1994-05-17-03), cl 4.7–6.3 mm, sampled together with the holotype.

*Caridina ablepsia* Guo & Jiang, 1992. Adult male (FU, 1989-05-23-01), tl 26.8 mm, cl 6.5 mm, rl 1.8 mm; a cave river at Xiaolongtong, Yunshun County, Hunan Province,China, 23 May 1989. Paratypes: 5 males (FU, 1989-05-23-02) cl 5.4–6.7 mm; 6 females (FU, 1989-05-23-03), cl 5.7–6.9 mm, sampled together with the holotype.

###### Diagnosis.

Rostrum short, slightly sloping downwards, usually reaching to the end of the 2^nd^ segment, occasionally reaching to the end of the 1^st^ segment or the end of the 3^rd^ segment of antennular peduncle, rostral formula 4–10+10–16/3–11. 1^st^ pereiopod carpus 0.77–0.83× as long as chela, 1.6–1.7× as long as high; chela 1.9–2.2× as long as broad; fingers 1.2–1.3× as long as palm. 2^nd^ pereiopod carpus 1.2–1.3× as long as chela, 4.7–6.1× as long as high; chela 2.2–2.9× as long as broad; fingers 1.6–2.3× as long as palm. 3^rd^ pereiopod propodus 3.8–4.1× as long as dactylus, with 9–11 thin spines on the posterior and lateral margins. 5^th^ pereiopod propodus 3.7–4.1 × as long as dactylus, with 11–13 thin spines on the posterior and lateral margins, dactylus terminating in one claw, with 38–44 spinules on flexor margin. Endopod of male 1^st^ pleopod extending to 0.45–0.50× exopod length, distal half usually curved posteriorly in the natural, occasionally not bent backwards, wider proximally, subrectangular, 2.4–2.7× as long as wide, appendix interna well developed, arising from distal 1/3 of endopod, reaching beyond end of endopod. Appendix masculina of male 2^nd^ pleopod rod-shaped, reaching to 0.51 length of endopod, appendix interna reaching to 0.93 length of appendix masculina. Uropodal diaeresis with 10–12 movable spinules. Eggs size (without eyespots) 0.67–0.82 × 1.29–1.38 mm, eggs size (containing embryos with eyes) 0.98–1.02 × 1.16–1.47 mm.

###### Description.

***Body*** (Fig. 5A–D): depigmented, slender and subcylindrical, medium-sized, males up to 22.7 mm tl, females up to 26.0 mm tl.

**Figure 3.**
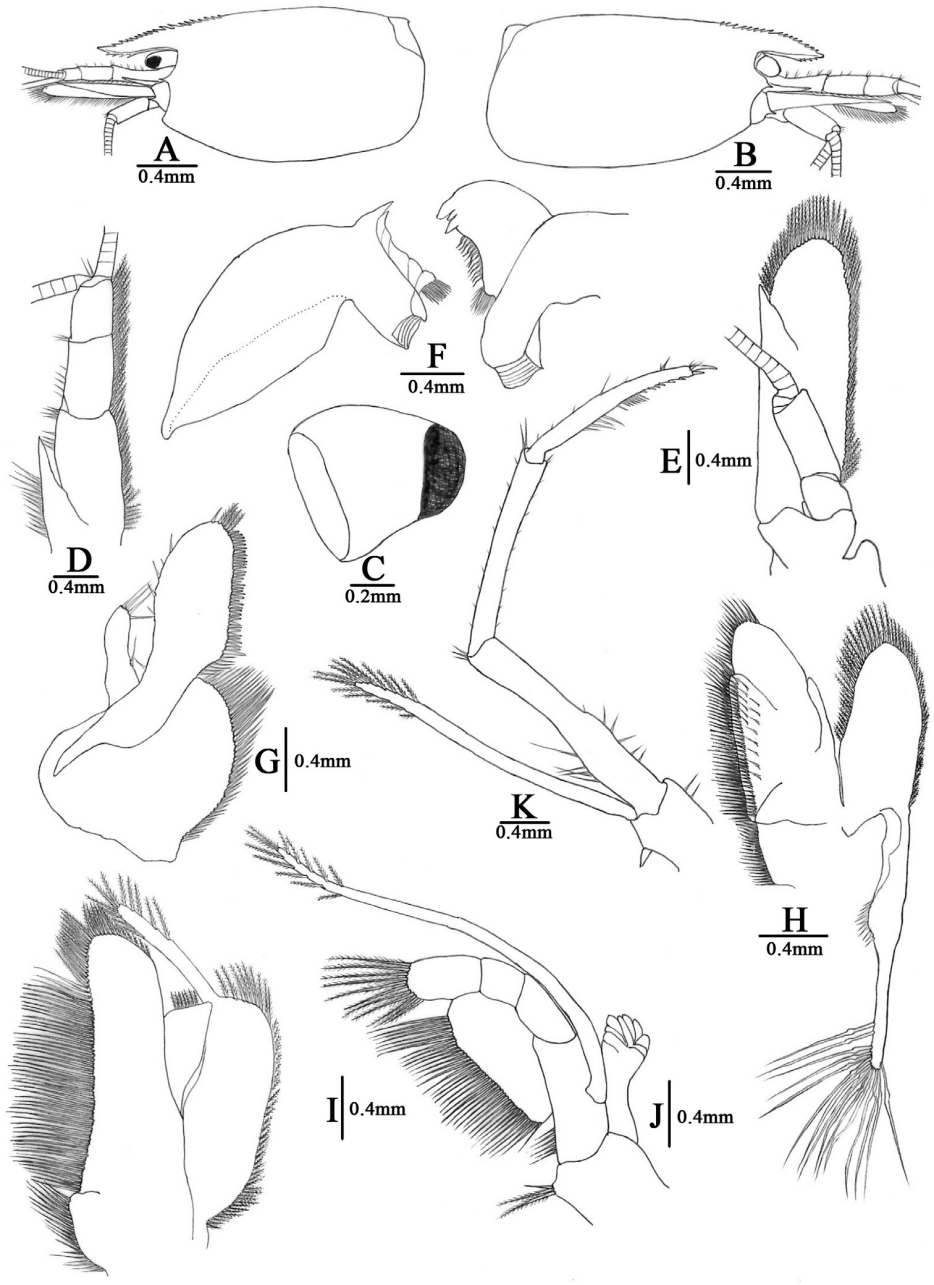
*Caridina sinanensis* sp. nov. **A, B** carapace and cephalic appendages, lateral view **C** eye **D** antennule **E** antenna **F** mandible **G** maxillula **H** maxilla **I** first maxilliped **J** second maxilliped **K** third maxilliped **A, C** holotype (FU, 2019-01-25-01) **D–K** paratype (FU, 2019-01-25-02) **B** paratype (FU, 2019-01-25-03).

***Rostrum*** (Fig. 3A, B): 0.25–0.47 of cl, reaching to the end of the 2^nd^ segment of antennular peduncle (75.8%, *N* =33) in large specimens, or to the end of the 1^st^ segment (15.2%), or to the end of the 3^rd^ segment of antennular peduncle (9.0%), straight, slightly sloping downwards; armed dorsally with 14–26 teeth, including 4–10 on carapace, ventrally with 3–11 teeth; lateral carina dividing rostrum into two unequal parts, continuing posteriorly to orbital margin.

***Eyes*** (Fig. 3A–C): small, partly reduced, with short stalk, cornea pigmentation variable, usually with pigment at centre of cornea, or totally absent (only one specimen).

***Carapace*** (Fig. 3A, B): smooth, glabrous; antennal spine acute, fused with inferior orbital angle; pterygostomial angle subrectangular, slightly protrude forward; pterygostomian spine absent.

***Antennule*** (Fig. 3D): peduncle short, reaching slightly short of scaphocerite; stylocerite short, reaching 0.75–0.88 length of basal segment; anterolateral angle reaching 0.20 length of the 2^nd^ segment; basal segment as long as combined length of the 2^nd^ and 3^rd^ segments, 2^nd^ segment as long as 0.53–0.61× of basal segment, 1.29–1.32× of the 3^rd^ segment; all segments with sub-marginal plumose setae.

***Antenna*** (Fig. 3E): peduncle about 0.53× as long as scaphocerite; scaphocerite 3.0–3.1× as long as wide, outer margin straight, asetose, ending in a strong subapical spine, inner and anterior margins with long plumose setae.

***Mandible*** (Fig. 3F): without palp, with well-developed incisor and molar processes; left and right mandible of similar size but differing in shape; left incisor process with single sharp tooth and a marginal transparent slice followed by patch of long setae, molar process strongly produced, ridged; right mandible incisor process with two long outer teeth and single short inner tooth, margin leading to molar process with 12 curving setae, followed by patch of long setae, molar process stout and with triturative surface.

***Maxillula*** (Fig. 3G): lower lacinia broadly rounded, with several rows of plumose setae; upper lacinia elongate, medial edge straight, with 36–42 strong spinules and simple setae; palp simple, longer than wide, slightly expanded distally, with four long simple setae.

***Maxilla*** (Fig. 3H): Scaphognathite well developed, tapering posteriorly, distally with regular row of long plumose setae and short marginal plumose setae continuing down proximal triangular process, furnished with numerous long plumose setae; upper and middle endites with marginal simple, denticulate and submarginal simple setae, distally with plumose setae; lower endite with long simple marginal setae; palp slightly shorter than the cleft of upper endite, wider proximally than distally, setose.

***First maxilliped*** (Fig. 3I): Palp broad with terminal plumose setae; caridean lobe broad, with marginal plumose setae; exopodal flagellum well developed, with distally marginal plumose setae; ultimate and penultimate segments of endopod indistinctly divided; medial and distal margins of ultimate segment with marginal and sub-marginal rows of simple, denticulate, and plumose setae; penultimate segments with marginal long plumose setae.

***Second maxilliped*** (Fig. 3J): endopodite ultimate and penultimate antennomeres fused, slightly concave, reflected against basal antennomeres, inner margin of ultimate, penultimate and basal segments with long setae of various types; exopod flagellum long, slender, with marginal plumose setae distally. Podobranchium is comb-like.

***Third maxilliped*** (Fig. 3K): endopod three-segmented, reaching slightly beyond scaphocerite; penultimate segment 0.87–0.92× of basal segment; distal segment as long as penultimate segment, ending in a large claw-like spine surrounded by simple setae, preceded by about 6–9 spines on distal third of posterior margin, proximally with a clump of long and short simple and serrate setae; exopod flagellum well developed, about a third the length of penultimate segment of endopod, distal margin with long plumose setae.

**Figure 4.**
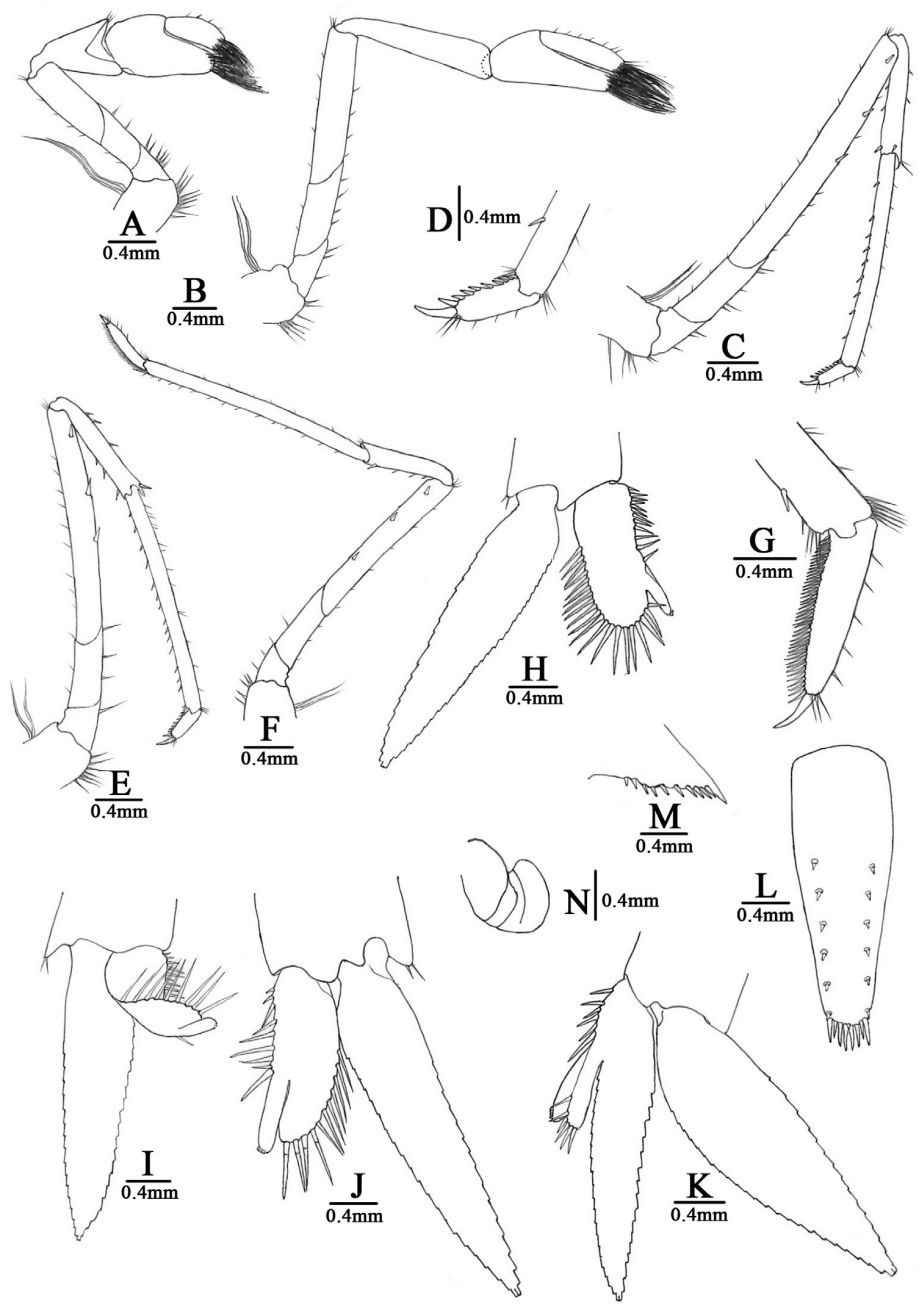
*Caridina sinanensis* sp. nov. **A** first pereiopod **B** second pereiopod **C** third pereiopod **D** dactylus of third pereiopod **E** fourth pereiopod **F** fifth pereiopod **G** dactylus of fifth pereiopod **H–J** first pleopod **K** second pleopod **L** telson **M** diaeresis of uropodal exopod **N** spermatophore **A–G, I–N** paratype (FU, 2019-01-25-02) **H** paratype (FU, 2019-01-25-04).

***First pereiopod*** (Fig. 4A): short, reaches end of eyes; chela length 1.9–2.2× breadth, 1.2–1.3× length of carpus; movable finger length 2.7–2.9× breadth, 1.2–1.3× length of palm, setal brushes well developed; carpus excavated disto-dorsally, length 1.6–1.7× breadth, 0.90–0.93× length of merus.

***Second pereiopod*** (Fig. 4B): reaches about end of 3^rd^ antennular peduncle segment, more slender and longer than first pereiopod; chela length 2.2–2.9× breadth, 0.79–0.85× length of carpus; movable finger length 3.8–4.4× breadth, and 1.6–2.3× length of palm, setal brushes well developed; carpus length 4.7–6.1× breadth, slightly excavated distally, 1.0–1.1× length of merus.

***Third pereiopod*** (Fig. 4C, D): reaches beyond end of scaphocerite; dactylus length 4.0–4.2× breadth, ending in prominent claw-like spine surrounded by simple setae, behind which bears 7–9 spines; propodus length 3.8–4.1× of dactylus, bearing 9–11 spinules on posterior margin, 11.2–12.2× breadth; carpus length 0.60–0.78× of propodus; merus length 1.9–2.1× of carpus, with about three large spines on the posterior margin.

***Fourth pereiopod*** (Fig. 4E): reaches end of 3^rd^ segment of antennular peduncle; dactylus length 4.0–4.2× breadth, ending in prominent claw-like spine surrounded by simple setae, behind which bears 7–8 spines; propodus length 3.9–4.3× of dactylus, bearing 11–16 spinules on posterior margin, 13.5–14.2× breadth; carpus length 0.53–0.62× of propodus; merus length 1.5–1.7× of carpus, with about three strong spines on the posterior margin.

***Fifth pereiopod*** (Fig. 4F, G): reaches the end of the 3^rd^ segment of antennular peduncle; dactylus length 4.9–5.4× breadth, ending in prominent claw-like spine surrounded by simple setae, behind which bears a comb-like row of 38–44 spines; propodus length 3.7–4.1× of dactylus, bearing 11–13 spinules on posterior margin, 16.6–17.6× breadth; carpus length 0.50–0.61× of propodus; merus length 1.4–1.5× of carpus, with about three strong spines on the posterior margin.

First four pereiopods with epipod. Branchial formula typical for genus.

***First pleopod*** (Fig. 4H–J): endopod of male subrectangular, distal half usually curved posteriorly in the natural, occasionally not bent backwards, wider proximally, length 0.45–0.50× exopod length, 2.4–2.7× proximal breadth, ending broadly rounded; inner margin slightly concave, bearing long spine-like setae, outer margin slightly convex or straightly, proximally 1/3 naked and distally 2/3 bearing nearly equal length spine-like setae; appendix interna well developed, arising from distal 1/3 of endopod, reaching to or beyond end of endopod, distally with cincinulli.

***Second pleopod*** (Fig. 4K): appendix masculina rod-shaped, reaching about 0.51× length of exopod, with numerous long spiniform setae proximally and distally, appendix interna well developed, almost same size as appendix masculina, reaching about 0.93× length of appendix masculina, distally with cincinulli.

***Telson*** (Fig. 4L): 0.34–0.47× of cl, shorter than the 6^th^ abdominal segment, 0.90–0.96× length of sixth abdominal segment, tapering posterior, with a median projection, dorsal surface with six pairs of stout movable spinules including the pair at poster lateral angles; posterior margin with four pairs of intermedial strong spiniform setae, sublateral pair shorter than lateral and inner pairs. Exopodite of the urpood bears a series of 10–12 movable spinules along the diaeresis, last one shorter than the lateral process.

**Female** carrying a number of 20–32 eggs, sized eggs 0.67–0.82 × 1.29–1.38 mm (without eyespots), and 0.98–1.02 × 1.16–1.47 mm (with eyespots).

**Figure 5.**
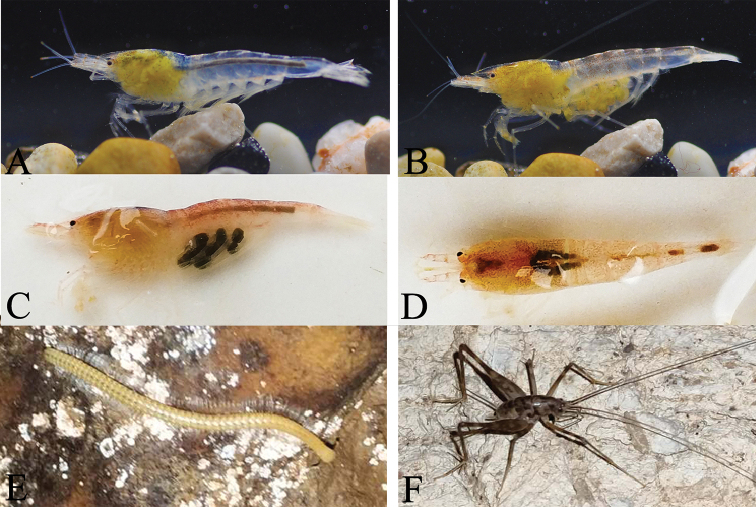
The cave dwelling organisms, colour in life **A–D***Caridina sinanensis* sp. nov. **E** a blind millipede **F** a camel cricket.

**Colouration** (Fig. 5A–D): body and appendages translucent white; eyes with black spot at centre of cornea; internal organs (gonads and hepatopancreas) whitish or yellowish; eggs in females yellowish or blackish.

###### Etymology.

*Caridina sinanensis* is named after Sinan County, where the type locality is located.

###### Remarks.

Six *Caridina* species lacking body pigmentation and having a small black spot on each eye are known from Chinese subterranean aquatic habitats: *C. acuta*, *C. alu*, *C. demenica*, *C. longshan*, *C. semiblesia*, and *C. sinanensis* These taxa can be readily separated into two groups by the rostrum shape and indentation. In the first group including *C. acuta*, *C. demenica*, and *C. semiblesia*, the rostrums are similarly lanceolate and short, with fewer teeth or unarmed. In the second group including *C. alu*, *C. longshan*, and *C. sinanensis* , the rostrums are long, reaching at least to the end of the 2^nd^ antennular segment, mostly beyond the end of scaphocerite, and armed with dorsal and ventral teeth. *Caridina. sinanensis* is morphologically close to *C. longshan* in sharing a similar spination pattern, the anterior region of endopod on the 1^st^ male pleopod folded backwards, and the variably pigmented cornea. *Caridina. sinanensis* can be distinguished from *C. longshan* by the relatively longer appendix interna on the appendix masculina of the 2^nd^ pleopod (reaching about 0.93 of appendix masculina vs 0.80 in *C. longshan*), the length of 6^th^ abdominal segment distinctly longer than the telson (vs same length of telson in *C. longshan*), and telson with posteromedian projection and lack of spinules on the surface of posterior telsonic spines (caudal spines) (vs lacking posteromedian projection and possessing spinules in *C. longshan*). *Caridina. sinanensis* can be easily separated from *C. alu* by its short rostrum (reaching to the end of the 2^nd^ antennular peduncle vs reaching to the end of scaphocerite in *C. alu*), the carpus of 1^st^ and 2^nd^ pereiopods are slender (length to breadth ratio 1.6–1.7 and 4.7–6.1 versus 1.3 and 2.6 in *C. alu*), the telson with posteromedian projection (vs lacking in *C. alu*), and male with completely different shape of the endopod of 1^st^ pleopods and appendix masculina of the 2^nd^ pleopods. *Caridina. sinanensis* sp. nov. also shows close similarity with *C. semiblesia* in the ratios of various segments of the 1^st^ and 2^nd^ pereiopods, and the shape of endopod of the 1^st^ pleopod in males. In addition to a longer rostrum that slopes downwards, *C. sinanensis* also differs from *C. semiblesia* in having the end of the palp of the 1^st^ maxilliped being broadly rounded and without a finger-like tip (vs ending in a finger-like tip in *C. semiblesia*), the smaller eggs (0.98–1.02 × 1.16–1.47 mm vs 1.05–1.15 × 1.37–1.71 mm), and the stout and long appendix interna of the appendix masculina on the 2^nd^ pleopod (appendix interna almost same size as appendix masculina and reaching about 0.93 of appendix masculina vs distinctly slender and reaching about 0.80 in *C. semiblesia*).

###### Molecular phylogenetic results.

**Figure 6.**
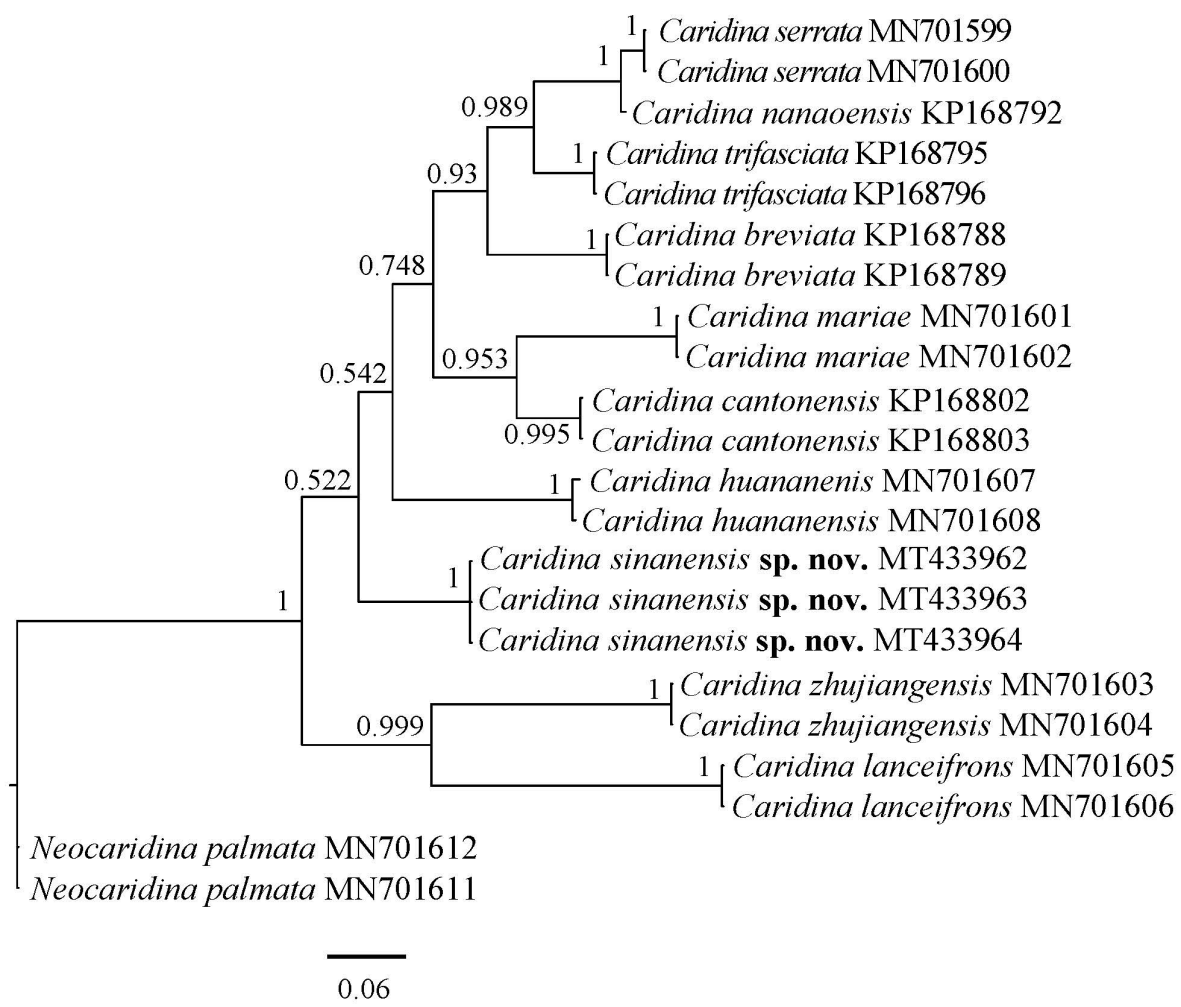
Bayesian inference (BI) tree of species of *Caridina* and outgroups (*Neocaridina*) based on COI gene. Support values at the nodes represent posterior probability.

**Figure 7.**
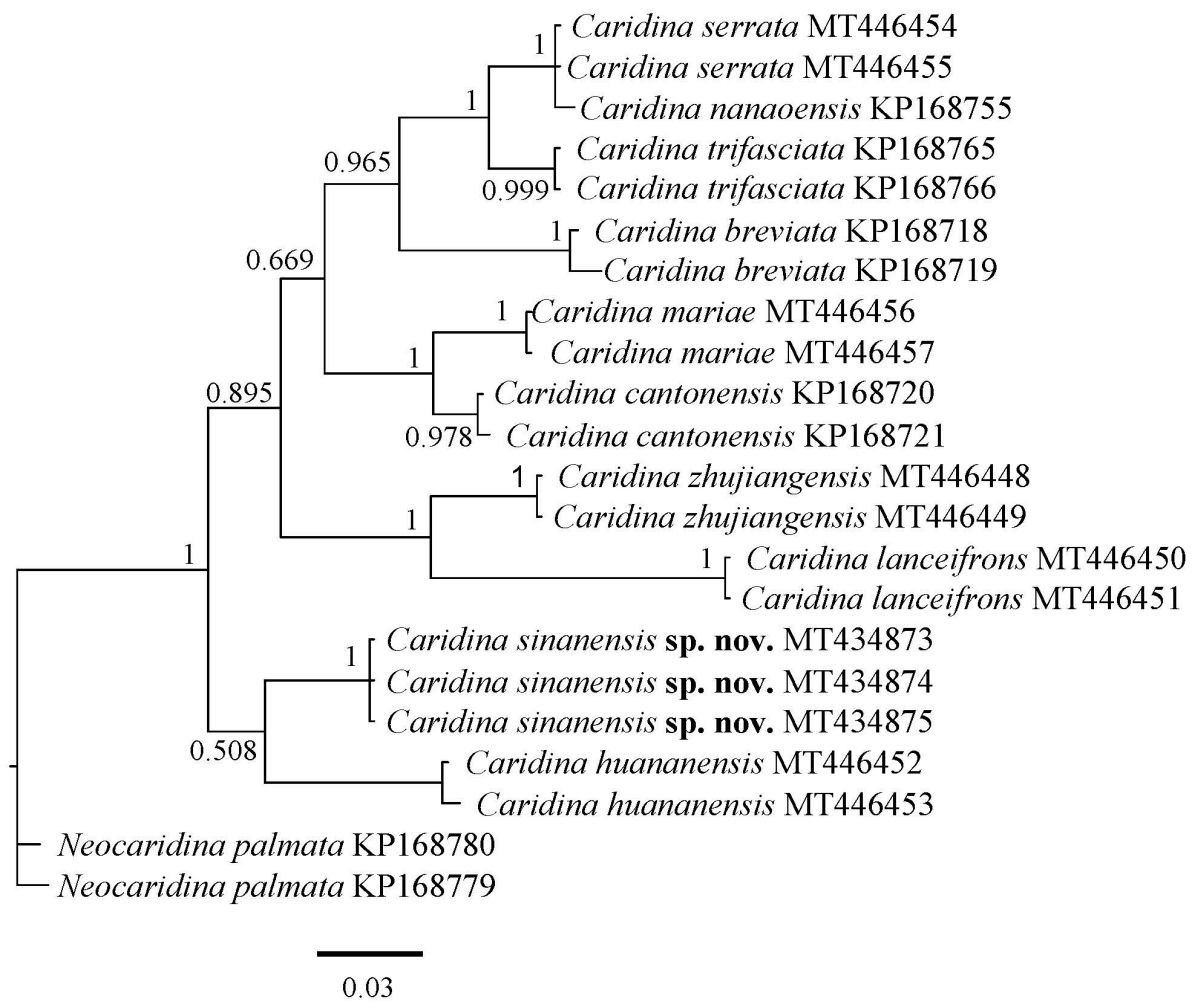
Bayesian inference (BI) tree of species of *Caridina* and outgroups (*Neocaridina*) based on 16S rRNA. Support values at the nodes represent posterior probability.

Including the GenBank sequences, we analysed 22 COI sequences and 22 16S rRNA sequences in total. The new sequencing results are corrected for 621~bp (COI) and 487~bp (16S) for subsequent analysis. Three specimens of *Caridina sinanensis* were used in the molecular phylogenetic analysis shown in Figures 6, 7. Specimens assigned to *C. sinanensis* formed a clade distinct from other species. And the tree topologies derived from COI and 16S rRNA analyses were basically congruent. *C. sinanensis* sp. nov. is well isolated from other nine *Caridina* with a sequence divergence of 15.3–26.7% (COI) and 7.2–11.2% (16S), respectively. According to Hebert et al. 2003, the genetic distances support the molecular-based description of *C. sinanensis* as a new species.

###### Ecological notes.

*Caridina sinanensis*, sp. nov. lives in an aphotic subterranean waterbody where the source of energy may come from allochthonous materials carried or washed into the cave, as there are particulates of vegetable debris in the water. Based on our observation on the shrimp’s feeding behavior and intestinal contents, this species feeds on detritus and microorganisms from the bottom sediments with its brush-tipped chelae and mouthparts, and the full intestine suggests that the foods are relatively abundant (Fig. 5C, D).

Leeches co-occurred with atyid shrimp in the subterranean waters, camel crickets were common on the cave rocks, especially in the dark zone, and a blind unpigmented species of millipede was found crawling along the rocks in the dark zone (Fig. 5E,F). Some trogloxene animals, such as bats and birds, were occasionally encountered in the cave entrance area.

In general, the populations of other cave-dwelling species were very small, while shrimp were moderately abundant. Competition for food and habitat seems insignificant, possibly because the groundwater was enriched with particulate organic matter and predators (such as leeches) were not abundant. Leeches are the natural enemies of the shrimp; they attach to the carapace, branchial chambers and appendages where they feed on the hemolymph of the shrimps. Parasitism certainly confers negative impact on populations of the new species, but accurate population data on the shrimp are lacking.

The sex ratio and reproduction season were preliminary inferred based on three sampling times. On 25 January 2019, 18 individuals were collected, including two adult males, 10 adult females (six ovigerous), and six subadult females. The sex ratio (male to female) is 1:8, and the percentage of ovigerous females is 60%. On 18 March 2019, 18 individuals were caught, including five adult males, 10 adult females (three ovigerous), and three subadult females. The sex ratio is 1:2.6, and the percentage of ovigerous females is 30%. In 18 February 2020, four individuals were caught including one adult male and three adult females (one ovigerous). The sex ratio is 1:3, and the percentage of ovigerous females is 33%. These results showed that the number of males was significantly less than females in the population from January to March. The causes responsible for the skewed sex ratio in favor of females may worth further study.

The ovigerous females comprised 60%, 33%, and 30% of mature females, respectively, in populations from January to March. One male carrying a spermatophore on the intermediate of the fifth walking legs was observed from specimens collected in January (Fig. 4N). This cave dwelling species has the highest number of reproductive individuals recorded during the winter and spring months, suggesting that the peak reproductive period occurs from January to March.

Females carried 20–32 eggs, size of undeveloped eggs (without eyespots) were 0.67–0.82 × 1.29–1.38 mm, size of developed eggs (containing embryos with eyes) 0.98–1.02 × 1.16–1.47 mm. The females of this species carry a small number of large eggs and produce eggs with a large amount of yolk and reduced number of larval stages. It is believed that abbreviated larval development may occurs in this species, larval direct development into benthic hatchlings that resemble miniature adults.

We are trying to understand embryonic development and hatching of this species. On 20 March 2019, five ovigerous females were transported to the laboratory for rearing, but unfortunately, after 7 days, the shrimps died.

###### Conservation.

Cave ecosystems are an invaluable resource, providing an ideal refuge for cave-dwelling species. Cave shrimp communities are particularly vulnerable to human disturbance, particularly groundwater pollution due to the local agricultural activities (fertilization, herbicide, and pesticide) and overexploitation (domestic usage and agricultural irrigation). These appear to be responsible for the pollution and degradation of subterranean habitat, but the extent of the impact is a little known. If groundwater become contaminated, local aquatic organisms certainly are at risk. Maintaining healthy groundwater shrimp communities requires the reduction of anthropogenic impacts, such as minimizing the use of agricultural pesticides, herbicides, and fertilizers by local farmers. It is suggested that the local government should ration the use of groundwater resources.

The Announcement of the Ministry of Agriculture and Rural Areas of China (CITES Appendix aquatic wild species of China, no. 69, 2018), fails to list freshwater shrimps in the CITES threatened categories. Since *Caridina sinanensis* is a new species, no conservation status has been assigned. According to the criteria listed in the IUCN Red List categories (IUCN 2019), *C. sinanensis* should be considered as a Critically Endangered species due to its exceptional rarity, restricted distribution in a single cave system, and the imminent threats from pollution. In order to better protect cave ecosystems, and their associated rare and threatened evolutionary relict fauna, it is critical and of great urgency to collect more baseline data on population and distribution patterns, delineate the importance and threatened status of cave fauna, and to devise corresponding conservation and management measures. Regular monitoring may be necessary to ensure populations are sustained in the face of further anthropogenic disturbances. Furthermore, cave biodiversity protection laws should be enacted as soon as possible.

**Table 2.** Environmental physicochemical parameters of the cave.

Environmental parameters	Unit	Light zone			Twilight zone			Dark zone		
		2019.1.25	2019.3.18	2020.2.15	2019.1.25	2019.3.18	2020.2.15	2019.1.25	2019.3.18	2020.2.15
Temperature(air)	°C	9.2	15.5	9.8	9.8	17.7	14.2	14.8	18.4	15.1
Temperature(water)	°C	/	/	/	20.1	20.2	20.2	20.2	20.3	20.2
Humidity(air)	%	76	78	80	90	92	93	94	96	97
pH(water)		/	/	/	6.6	6.5	6.7	6.6	6.6	6.7
oxygen(air)	%	28	27	29	23	25	24	21	22	22
Hydrogen sulfide(air)	mg/kg	0.45	0.48	0.44	0.37	0.40	0.39	0.21	0.25	0.22
Carbon monoxide(air)	mg/kg	17.0	17.2	17.2	16.9	16.1	14.9	11.5	12.2	11.7
Carbon dioxide(air)	mg/kg	270	273	280	270	274	283	355	348	367
Dissolved oxygen(water)	mg/L	/	/	/	8.3	8.0	8.7	8.3	8.0	8.7

## Discussion

*Caridina sinanensis* sp. nov. is not blind, as the eyes still contain considerable pigmentation, although we found one male specimen lacking pigments in its cornea (Fig. 4B). The body is almost transparent but still shows a yellowish colour. Such presumably derived structures of the eyes and body coloration might represent an adaptation to aphotic cave environments. Therefore, the species has successfully adapted to the subterranean environment and can be considered a stygobiotic atyid.

The freshwater atyid species associated with caves are relatively numerous in China. The number of subterranean atyids recorded now totals 24, but only 13 species can be regarded as real troglobionts. Of these, five have reached the advanced stage in which the faceted cornea of the eyes is absent, namely: *Caridina alba*, *C. ablepsia*, *C. caverna*, *Mancicaris sinensis*, and *Typhlocaridina lanceifrons*. Eight species with reduced eyes but presence of pigmented cornea are: *C. acuta*, *C. alu*, *C. demenica*, *C. longshan*, *C. semiblesia*, *T. liui*, *T. semityhplata*, and *C. sinanensis* sp. nov. In order to contribute to the management of the vulnerable subterranean ecosystems and their highly specialized endemic stygofauna, there is an urgent need to accelerate scientific research, including but not limited to collecting the much-needed information on taxonomy, life history, ecology, and distribution, and to design monitoring programs of subterranean species, especially for those that are under the most intense threats from anthropogenic factors.

As molecular analysis has become a crucial step towards resolving taxonomic problems, delimitations, and the real biodiversity of atyids, a combined morphological and molecular data is necessary for the description of a new taxa. Ideally, this process will involve to comprehensive information on color, physiology, and ecology. In Chinese karst landforms, as many caves are explored and the molecular identification applied, the species list will increase with future surveys.

## Supplementary Material

XML Treatment for Caridina sinanensis
